# Clinical Significance of Community- and Healthcare-Acquired Carbapenem-Resistant Enterobacteriaceae Isolates

**DOI:** 10.1371/journal.pone.0151897

**Published:** 2016-03-21

**Authors:** Hung-Jen Tang, Cheng-Fang Hsieh, Ping-Chin Chang, Jyh-Jou Chen, Yu-Hsiu Lin, Chih-Cheng Lai, Chien-Ming Chao, Yin-Ching Chuang

**Affiliations:** 1 Department of Medicine, Chi Mei Medical Center, Tainan, Taiwan; 2 Department of Health and Nutrition, Chia Nan University of Pharmacy and Science, Tainan, Taiwan; 3 Division of Geriatrics and Gerontology, Department of Internal Medicine, Kaohsiung Medical University Hospital, Kaohsiung Medical University, Kaohsiung, Taiwan; 4 Department of Neurology, Kaohsiung Medical University Hospital, Kaohsiung Medical University, Kaohsiung, Taiwan; 5 Department of Internal Medicine, Chi Mei Medical Center, Liouying, Tainan, Taiwan; 6 The Committee of Infection Control, Chi Mei Medical Center, Liouying, Tainan, Taiwan; 7 Department of Intensive Care Medicine, Chi Mei Medical Center, Liouying, Tainan, Taiwan; 8 Department of Nursing, Min-Hwei College of Health Care Management, Tainan, Taiwan; 9 Department of Medical Research, Chi Mei Medical Center,Tainan, Taiwan; Beijing Institute of Microbiology and Epidemiology, CHINA

## Abstract

This study was conducted to investigate the clinical significance, manifestations, microbiological characteristics and outcomes of carbapenem-resistant Enterobacteriaceae (CRE) isolates, and compare the clinical features of community- and healthcare-acquired CRE isolates. A total of 78 patients were identified to have CRE. *Klebsiella pneumoniae* was the most common pathogens (n = 42, 53.8%), followed by *Enterobacter cloacae* (n = 24, 30.8%), and *Escherichia coli* (n = 11, 14.1%). Most of the patients acquired CRE from healthcare settings (n = 55, 70.5%), and other cases got CRE from community settings (n = 23, 29.5%). Nine cases (11.5%) were classified as CRE colonization. Among the remaining 69 cases of CRE infections, pneumonia (n = 28, 40.6%) was the most common type of infections, followed by urinary tract infection (n = 24, 34.8%), and intra-abdominal infection (n = 16, 23.2%). The patients acquired CRE from community settings were more likely to be elderly, female, and had more urinary tract infections than from healthcare settings. In contrast, the patients acquired CRE from healthcare settings had more intra-abdominal infections, intra-abdominal surgery, and presence of indwelling device than from community settings. In conclusion, community-acquired CRE are not rare, and their associated clinical presentations are different from healthcare-acquired CRE.

## Introduction

Antibiotic-resistant bacteria are difficult to treat and can be associated with high morbidity and mortality. Therefore, they pose a great threat to public health. There is no exception for Enterobacteriaceae, and their resistances to broad-spectrum antimicrobials, such as extended-spectrum cephalosporins, have rapidly increased. For a long time, carbapenems have been considered as an important antibiotic for the treatment of Enterobacteriaceae; however, carbapenem-resistance among Enterobacteriaceae is emerging recently. Till now, carbapenem-resistant Enterobacteriaceae (CRE) have become a global issue [1–7]. In Taiwan, the prevalence of CRE remained low in spite of its increases in recent years. Most of studies limited their focus on a single bacterial species or a single type of infectious disease, which could not show the whole picture of CRE [8–15]. In addition, although CRE are initially considered as hospital-acquired pathogens, community-acquired CRE are also noted [9]. However, the knowledge about community-acquired CRE is limited. Therefore, this study was conducted to investigate the clinical significances, manifestations, microbiological characteristics and outcomes of CRE isolates, and compare the clinical features of community- and healthcare-acquired CRE isolates.

## Methods

### Setting

This study was conducted at the Chi Mei Medical Center, a 900-bed with 63 adult intensive care unit (ICU) beds in southern Taiwan. Patients with cultures positive for CRE during the period January 2015 to July 2015 were identified from the hospital’s computerized database. The medical records of all patients with positive isolates of CRE were retrospectively reviewed. Demographic data including age, gender, underlying conditions including history of immunosuppressant drug use, diabetes mellitus, liver cirrhosis, chronic kidney disease, malignancy, the use of medical devices, and prior medical examinations were collected. In addition, antimicrobial susceptibility results, and outcomes were collected. The data were collected on a routine basis and the analyses were carried out retrospectively. Therefore, no informed consent was required and it was specifically waived by Institutional Review Board. Ethics approval was obtained from Institution Review Board of Chi Mei Medical Center.

### Definitions

If CRE were isolated from the patient who had recently been hospitalized for > 48 hours in the previous two weeks or resided in long-term care facilities, they were defined as healthcare-acquired CRE. Otherwise, patients were considered to obtain CRE in community settings. The diagnosis of infection focus was made based on clinical, bacteriological, and radiological investigations [16]. As previous study [16], catheter-related bloodstream infection was defined as a positive semi-quantitative tip culture (≥ 15 colony-forming units [CFU]), bacteremia, and/or high clinical suspicion; pneumonia was defined as a positive culture for CRE in purulent sputum samples and the presence of newly developed lung infiltrates; urinary tract infection (UTI) was defined as positive urine culture with growth of ≥ 10^5^ CFU/ml and pyuria. Mortality was defined as death from all causes during the episode of hospitalization. The definition of infection or colonization was followed the guidelines published by the Centers for Disease Control and Prevention. Extended-spectrum cephalosporins included ceftriaxone, flomoxef, ceftazidime, and cefpirome. Extended-spectrum β-lactam/β-lactamase inhibitor combinations included amoxicillin/clavulanate and piperacillin/tazobactam. Carbapenems included imipenem, meropenem, and ertapenem. Fluoroquinolones included ciprofloxacin, moxifloxacin and levofloxacin. Glycopeptide included vancomycin and teicoplanin.

### Microbiological investigation

Enterobacteriacae isolates were identified by conventional biochemical tests and by two commercial identification kits, Api20NE (bioMerieux, Marcy I`Etoile, France) and the Phoenix System (Becton Dickson, Sparks, MD). Isolates were classified as susceptible or resistant (including an intermediate category) by broth microdilution methods according to Clinical and Laboratory Standards Institute (CLSI) guidelines [17, 18]**.** The β-lactam agents tested included ampicillin, amoxicillin/clavulanate, piperacillin/tazobactam, cefazoline, cefuroxime, ceftazidime, ceftriaxone, flomoxef, and two carbapenems included ertapenem, and imipenem. Non β-lactam agents tested included gentamicin, amikacin, ciprofloxacin, and tigecycline. CRE were defined as Enterobacteriacae isolates resistant to imipenem or ertapenem.

### Statistical analysis

Continuous variables are expressed as the mean ± standard deviation. Continuous variables were compared using the Wilcoxon rank-sum test or Student’s independent *t* test, as appropriate. Categorical variables were compared using the chi-square test or Fisher’s exact test. All statistical analyses were conducted using the statistical package SPSS for Windows (Version 11.0, SPSS, Chicago, Il, USA).

## Results

During the study period, a total of 78 patients were identified to have CRE isolates from clinical specimens (S1 Table). The mean age of the patients was 71.3 years, and 52 (66.7%) patients were classified as elderly patients ≥ 65 years old. Men comprised 61.5% of patients. *Klebsiella pneumoniae* was the most common pathogens (n = 42, 53.8%), followed by *Enterobacter cloacae* (n = 24, 30.8%), *Escherichia coli* (n = 11, 14.1%) and *Proteus mirabilis* (n = 1, 1.3%). Most of the patients acquired CRE from healthcare settings (n = 55, 70.5%), and other cases got CRE from community settings (n = 23, 29.5%). Among healthcare settings, general ward (n = 37), especially hematological department, was the most common site of CRE acquisition, followed by ICU (n = 15), and nursing home (n = 3). Nine cases (11.5%) were classified as CRE colonization, including eight isolates from sputum, and one from urine sample. Among the remaining 69 cases of CRE infections, pneumonia (n = 28, 40.6%) was the most common types of infections, followed by urinary tract infection (n = 24, 34.8%), intra-abdominal infection (n = 16, 23.2%) and central line-associated infection (n = 1, 1.45%). Additionally, 11 cases had ventilator-associated pneumonia. Cancer (n = 34, 43.6%) was the most common underlying diseases, followed by diabetes mellitus (n = 28, 35.9%), chronic kidney diseases (n = 17, 21.8%), and liver cirrhosis (n = 10, 12.8%). Overall, a total of 65 patients (83.3%) had underlying immunocompromised conditions either due to underlying diseases or treatment. The uses of immunosuppressant and steroid were found in 29.5% and 25.6% of cases, respectively. Sixty-nine (88.5%) patients had ever received broad-spectrum antibiotics before acquiring CRE isolates, and 42 patients had received prior antibiotic with carbapenem. More than half of patients ever received extended-spectrum cephalosporin and carbapenem before the episode. More than 40% of patients had received extended spectrum β-lactam/β-lactamase inhibitor, quinolone or glycopeptide. The overall in-hospital mortality was 14 (17.9%).

The comparisons between community-acquired and healthcare-acquired CRE isolates are summarized in Table 1. The patients acquired CRE from community settings were more likely to be elderly, female, and had more urinary tract infections than from healthcare settings. In contrast, the patients acquired CRE from healthcare settings had more intra-abdominal infections, intra-abdominal surgery, and presence of medical device, such as nasogastric tube, Foley tube, and central venous catheter than from community settings.

**Table 1:** Clinical manifestations of 78 patients with clinical isolations of carbapenem-resistant *Enterobacteriacae* that were acquired from community or healthcare setting.

Variable	No. (%) of community–acquired (n = 23)	No. (%) of healthcare-acquired (n = 66)	P value
Age ≥ 65	20 (87.0)	32 (58.2)	0.028
Male (%)	9 (39.1)	39 (70.9)	0.018
Pathogens			
*K*. *pneumoniae*	13 (56.5)	29 (52.7)	0.957
*E*. *cloacae*	5 (21.7)	19 (34.5)	0.396
*E*. *coli*	4 (17.4)	7 (12.7)	0.852
*P*. *mirabilis*	1 (4.3)	0 (0.0)	0.661
Clinical significance			0.154
Colonization	5 (21.7)	4 (7.3)	
Infection	18 (78.3)	51 (92.7)	
Pneumonia	7 (38.9)	21 (41.2)	0.914
Urinary tract infection	11 (61.1)	13 (25.5)	0.015
Intra-abdominal infections	0 (0.0)	16 (31.4)	0.017
Central line-associated infection	0 (0.0)	1 (2.0)	0.595
Bacteremia	0 (0.0)	6 (10.9)	0.237
Underlying diseases/conditions			
Cancer	6 (26.1)	28 (50.9)	0.078
Diabetes mellitus	11 (47.1)	17 (30.9)	0.270
Chronic kidney disease	7 (30.4)	10 (18.2)	0.374
Liver cirrhosis	3 (13.0)	7 (12.7)	0.737
Steroid use	5 (21.7)	15 (27.3)	0.816
Immunosuppressant use	3 (13.0)	20 (36.4)	0.073
Receive total parenteral nutrition	0 (0.0)	5 (9.1)	0.323
Intra-abdominal surgery	0 (0.0)	12 (21.8)	0.037
Procedure within 3 months			
Esophagogastroduodenoscopy	2 (8.7)	19 (34.5)	0.039
Bronchoscopy	2 (8.7)	3 (5.5)	0.985
Colonoscopy	0 (0.0)	3 (5.5)	0.614
In-hospital mortality	4 (17.4)	10 (18.2)	0.811

### Microbiology findings

The results of in vitro susceptibility testing to various antimicrobial agents against CRE are shown in Table 2. Half of the CRE isolates were extended-spectrum β-lactamase (ESBL)-producer. All of the isolates were not susceptible to 1^st^ and 2^nd^ generation cephalosporin, ceftazidime, ampicillin, and amoxicillin/clavulanate. Although 97% of CRE isolates were not susceptible to ertapenem, less than one-third were not susceptible to imipenem. Amikacin showed good in vitro activity against almost 80% of clinical isolates.

**Table 2:** Antibiotic non-susceptible patterns.

Antibiotic	Number (%) of non-susceptible rate			
	All isolates (n = 78)	*K*. *pneumoniae* (n = 42)	*E*. *cloacae* (n = 24)	*E*. *coli* (n = 11)
ESBL-producer	39 (50.0)	33 (78.6)	0 (0.0)	5 (45.5)
Cefazolin	78 (100.0)	42 (100.0)	24 (100.0)	11 (100.0)
Cefuroxime	78 (100.0)	42 (100.0)	24 (100.0)	11 (100.0)
Ceftriaxone	76 (97.4)	40 (95.2)	24 (100.0)	11 (100.0)
Cefatazidime	78 (100.0)	42 (100.0)	24 (100.0)	11 (100.0)
Flomoxef	70 (89.7)	37 (88.1)	24 (100.0)	9 (81.8)
Gentamicin	42 (53.8)	24 (57.1)	11 (45.8)	6 (54.5)
Amikacin	16 (20.5)	11 (26.2)	2 (8.3)	2 (18.2)
Ampicillin	78 (100.0)	42 (100.0)	24 (100.0)	11 (100.0)
Augmentin	78 (100.0)	42 (100.0)	24 (100.0)	11 (100.0)
Piperacillin/tazobactam	69 (88.5)	37 (88.1)	22 (91.7)	10 (90.9)
Ciprofloxacin	71 (91.0)	42 (100.0)	18 (75.0)	10 (90.9)
Ertapenem	76 (97.4)	42 (100.0)	22 (91.7)	11 (100.0)
Imipenem	25 (32.1)	12 (28.6)	9 (37.5)	3 (27.3)
Tigecycline	42 (53.8)	24 (57.1)	17 (70.8)	0 (0.0)

ESBL = Extended-spectrum β-lactamases

## Discussion

This six-month study that enrolled 78 patients with clinical isolates of CRE had several significant findings. Among these 78 cases with CRE isolates, about 30% of them acquired CRE from community settings. It is higher than previous study [9] at a teaching hospital in Taiwan during 2010, which showed that 12% of 117 CRE isolates were community-acquired, and another surveillance investigation [6] in community hospitals in the southeastern United States from 2008 to 2012, which revealed that 17 (6%) of 305 CRE isolates were community-acquired. Thus, these findings suggest CRE have disseminated to the community, and clinicians should consider CRE as possible pathogens causing community-acquired infections.

Previous studies [19–24] have identified several risk factors associated with acquisition of CRE, including exposure to antibiotics (such as carbapenem and quinolones), healthcare exposure, presence of indwelling devices (such as central line, urinary catheter, endotracheal tube and feeding tube), use of mechanical ventilator, and comorbidities. Although the present work had the similar findings that most of cases had variable immunocompromised conditions or risk factors, such as prior exposure to broad-spectrum antibiotics, and recent invasive procedure or examinations, we also found that the clinical features of community-acquired and healthcare-acquired CRE were significantly different. Most cases of community-acquired CRE were elderly and the most common type of clinical infection was urinary tract infection in this specific populations. However, the case number in this study was limited. Further large-scale study is warranted to investigate the epidemiological characteristics of community-acquired CRE.

In our study, *K*. *pneumoniae* was the most common species of CRE, followed by *E*. *cloacae*, and *E*. *coli*. It is consistent with a previous study [14] of 1135 CRE isolates in Taiwan, which reported that the most common species were *K*. *pneumoniae* (n = 577, 50.8%), followed by *E*. *cloacae* complex (n = 267, 23.5%), and *E*. *coli* (n = 145, 12.8%), and another study [9] of 117 carbapenem-nonsusceptible Enterobacteriaceae (CNSE) at a teaching hospital in Taiwan, which showed that the most common organisms were *K*. *pneumoniae* (58.1%), *E*. *cloacae* (26.5%), and *E*. *coli* (9.4%). However, the microbiologic profiles of CRE in Taiwan are a little different from those in other countries. For example, CRE were commonly seen in *K*. *pneumoniae* (42.2%), *E*. *coli* (24.3%) and *E*. *cloacae* (17.2%) among the 268 isolates in a Singapore’s study [25]. In a recent study [26] of Asia countries or regions, *Klebsiella* spp. and *E*. *coli* account for the largest proportion of CRE, namely 39.3% and 22.0%, and then followed by *Serratia* spp. (19.8%), *Enterobacter* spp. (13.0%), *Proteus* spp. (4.0%), and *Citrobacter* spp. (2.0%). All of these findings indicate that the bacterial distribution of CRE isolates may vary according to different sites and suggest that every site or region should perform surveillance investigation to establish its own epidemiological characteristics.

In line with a previous study [14], most of CRE isolates in the present work were resistant to ertapenem, but still susceptible to imipenem. Moreover, each bacterial species had its own antibiotic resistant pattern according to the in vitro tests. Although most of CRE isolates were resistant to many antibiotics, amikacin still showed good in vitro activity against CRE in our study. It may indicate that aminoglycoside would be a good drug of choice for combination antimicrobial therapy for CRE infections.

Our study has one major limitation. The case number is limited, especially for community-acquired CRE cases, and this limitation may be due to the low prevalence of community-acquired CRE in Taiwan. In addition, the clinical isolates in this retrospective study was not kept for further investigation the mechanism of carbapenem resistance. Moreover, we did not perform fingerprinting tests to identify possible clonal spread. Although we used epidemiological investigation to clarify this issue and no clonal spread was detected, further study using advanced molecular method is warranted for better understand the clinical characteristics of each CRE isolates.

In conclusion, community-acquired CRE are not rare, and their clinical presentations are different from healthcare-acquired CRE. Active surveillance of CRE should be indicated, even in the community setting.

## Supporting Information

S1 Table Clinical characteristics of the study patients. (DOCX)
