# Supplementary material for: Clinical Significance of Community- and Healthcare-Acquired Carbapenem-Resistant Enterobacteriaceae Isolates
Source: PLoS One. 2016 Mar 21;11(3):e0151897. doi: 10.1371/journal.pone.0151897 (PMC4801408; doi:10.1371/journal.pone.0151897)
Supplement: S1 Table — (DOCX) [file pone.0151897.s001.docx]

Supplemental table 1. Clinical characteristics of the study patients

| Case number | Site of acquisition | Clinical significance | Immunocompromised condition | Initial appropriate antibiotic* | In-hospital mortality |
| --- | --- | --- | --- | --- | --- |
| 1 | General ward | Central line-associated infection | Yes | Yes | No |
| 2 | General ward | Intra-abdominal infection | Yes | Yes | No |
| 3 | General ward | Intra-abdominal infection | Yes | Yes | No |
| 4 | ICU | Intra-abdominal infection | No | Yes | No |
| 5 | General ward | Intra-abdominal infection | Yes | Yes | No |
| 6 | General ward | Intra-abdominal infection | Yes | No | No |
| 7 | General ward | Intra-abdominal infection | No | Yes | No |
| 8 | General ward | Intra-abdominal infection | Yes | No | No |
| 9 | ICU | Intra-abdominal infection | No | Yes | No |
| 10 | General ward | Intra-abdominal infection | Yes | No | No |
| 11 | General ward | Intra-abdominal infection | Yes | Yes | No |
| 12 | General ward | Intra-abdominal infection | Yes | Yes | No |
| 13 | General ward | Intra-abdominal infection | Yes | Yes | No |
| 14 | General ward | Intra-abdominal infection | Yes | No | No |
| 15 | General ward | Intra-abdominal infection | Yes | Yes | No |
| 16 | General ward | Intra-abdominal infection | Yes | Yes | Yes |
| 17 | ICU | Intra-abdominal infection | Yes | No | Yes |
| 18 | Community | Pneumonia | Yes | Yes | No |
| 19 | General ward | Pneumonia | Yes | Yes | No |
| 20 | ICU | Pneumonia | Yes | No | No |
| 21 | Community | Pneumonia | Yes | No | No |
| 22 | Nursing home | Pneumonia | Yes | No | No |
| 23 | General ward | Pneumonia | Yes | Yes | No |
| 24 | General ward | Pneumonia | Yes | No | No |
| 25 | ICU | Pneumonia | Yes | No | No |
| 26 | General ward | Pneumonia | Yes | No | No |
| 27 | General ward | Pneumonia | Yes | Yes | No |
| 28 | ICU | Pneumonia | Yes | No | No |
| 29 | General ward | Pneumonia | No | No | No |
| 30 | Community | Pneumonia | Yes | No | No |
| 31 | ICU | Pneumonia | Yes | Yes | No |
| 32 | ICU | Pneumonia | Yes | Yes | No |
| 33 | Community | Pneumonia | Yes | No | No |
| 34 | Community | Pneumonia | Yes | No | No |
| 35 | ICU | Pneumonia | Yes | No | No |
| 36 | General ward | Pneumonia | Yes | No | No |
| 37 | General ward | Pneumonia | Yes | Yes | No |
| 38 | ICU | Pneumonia | No | Yes | Yes |
| 39 | Community | Pneumonia | Yes | Yes | Yes |
| 40 | General ward | Pneumonia | Yes | No | Yes |
| 41 | General ward | Pneumonia | Yes | Yes | Yes |
| 42 | General ward | Pneumonia | Yes | No | Yes |
| 43 | Community | Pneumonia | No | No | Yes |
| 44 | General ward | Pneumonia | Yes | No | Yes |
| 45 | General ward | Pneumonia | Yes | No | Yes |
| 46 | General ward | Urinary tract infection | Yes | Yes | No |
| 47 | General ward | Urinary tract infection | Yes | Yes | No |
| 48 | Community | Urinary tract infection | Yes | No | No |
| 49 | Community | Urinary tract infection | Yes | No | No |
| 50 | ICU | Urinary tract infection | Yes | No | No |
| 51 | Community | Urinary tract infection | No | No | No |
| 52 | General ward | Urinary tract infection | No | No | No |
| 53 | ICU | Urinary tract infection | Yes | Yes | No |
| 54 | Community | Urinary tract infection | No | No | No |
| 55 | Nursing home | Urinary tract infection | No | No | No |
| 56 | General ward | Urinary tract infection | Yes | Yes | No |
| 57 | Community | Urinary tract infection | Yes | No | No |
| 58 | Nursing home | Urinary tract infection | Yes | Yes | No |
| 59 | General ward | Urinary tract infection | Yes | No | No |
| 60 | General ward | Urinary tract infection | Yes | Yes | No |
| 61 | Community | Urinary tract infection | Yes | No | No |
| 62 | Community | Urinary tract infection | No | No | No |
| 63 | Community | Urinary tract infection | No | No | No |
| 64 | General ward | Urinary tract infection | No | No | No |
| 65 | Community | Urinary tract infection | Yes | Yes | No |
| 66 | ICU | Urinary tract infection | Yes | Yes | Yes |
| 67 | ICU | Urinary tract infection | Yes | Yes | Yes |
| 68 | Community | Urinary tract infection | Yes | No | Yes |
| 69 | Community | Urinary tract infection | Yes | No | Yes |
| 70 | ICU | Colonization | Yes |  | No |
| 71 | Community | Colonization | Yes |  | No |
| 72 | General ward | Colonization | Yes |  | No |
| 73 | General ward | Colonization | Yes |  | No |
| 74 | Community | Colonization | Yes |  | No |
| 75 | Community | Colonization | Yes |  | No |
| 76 | General ward | Colonization | Yes |  | No |
| 77 | Community | Colonization | Yes |  | No |

* Initial appropriate use of empirical antibiotics was considered as the empirical usage of antimicrobial agents that were susceptible in vitro against carbapenem-resistant Enterobacteriaceae Isolates isolates.
